# Assessment of the Level of Accumulation of the dIFN Protein Integrated by the Knock-In Method into the Region of the Histone H3.3 Gene of *Arabidopsis thaliana*

**DOI:** 10.3390/cells10082137

**Published:** 2021-08-19

**Authors:** Natalya V. Permyakova, Tatyana V. Marenkova, Pavel A. Belavin, Alla A. Zagorskaya, Yuriy V. Sidorchuk, Elena A. Uvarova, Vitaliy V. Kuznetsov, Sergey M. Rozov, Elena V. Deineko

**Affiliations:** Federal Research Center Institute of Cytology and Genetics, Siberian Branch of Russian Academy of Sciences, pr. Lavrentieva 10, 630090 Novosibirsk, Russia; marenkova@bionet.nsc.ru (T.V.M.); belvin@bionet.nsc.ru (P.A.B.); zagorska@bionet.nsc.ru (A.A.Z.); sidorch@bionet.nsc.ru (Y.V.S.); uvarova@bionet.nsc.ru (E.A.U.); vitkuz@ngs.ru (V.V.K.); rozov@bionet.nsc.ru (S.M.R.); deineko@bionet.nsc.ru (E.V.D.)

**Keywords:** Arabidopsis, cell culture, gene editing, knock-in, histone H3.3 gene, deltaferon

## Abstract

Targeted DNA integration into known locations in the genome has potential advantages over the random insertional events typically achieved using conventional means of genetic modification. We investigated the possibility of obtaining a suspension cell culture of *Arabidopsis thaliana* carrying a site-specific integration of a target gene encoding modified human interferon (dIFN) using endonuclease Cas9. For the targeted insertion, we selected the region of the histone H3.3 gene (*HTR5*) with a high constitutive level of expression. Our results indicated that Cas9-induced DNA integration occurred with the highest frequency with the construction with donor DNA surrounded by homology arms and Cas9 endonuclease recognition sites. Among the monoclones of the four cell lines with knock-in studied, there is high heterogeneity in the level of expression and accumulation of the target protein. The accumulation of dIFN protein in cell lines with targeted insertions into the target region of the *HTR5* gene does not statistically differ from the level of accumulation of dIFN protein in the group of lines with random integration of the transgene. However, one among the monoclonal lines with knock-in has a dIFN accumulation level above 2% of TSP, which is very high.

## 1. Introduction

Recombinant proteins are foreign proteins produced in various expression systems. They are mainly used as pharmaceuticals for diagnostics, for the vaccination of humans or animals, and as drugs or monoclonal antibodies [1]. The ever-growing demand for recombinant proteins stimulates the development of various expression systems for the production of these proteins that meet the existing stringent standards. Today, recombinant proteins are mainly produced in traditional prokaryotic and eukaryotic systems such as *Escherichia coli*, several yeast species, and mammalian cell cultures. More than 50% of all pharmaceutical recombinant proteins are synthesized in mammalian cells [2,3]. Mammalian cells are necessary for the production of complex proteins because bacteria cannot form disulfide bonds efficiently and neither bacteria nor yeast can add human-like post-translational modifications (PTMs) in the secretory pathway [4]. Protein production in mammalian cells is expensive due to the high cost of the culture media components. Moreover, some recombinant proteins, such as immunotoxins or regulatory proteins, are toxic to mammalian cells. Production of biopharmaceuticals in plant expression systems is becoming a promising alternative to existing platforms based on mammalian and bacterial cells [2,5,6]. The cultivation of plant cells under controlled conditions of bioreactors ensures the production of high-quality protein according to GMP standards. The advantages of plant expression systems are rapid cell growth, low cost of nutrient media components, the low risk of contamination with animal pathogens, as well as the possibility of obtaining recombinant proteins with a modified human-type glycosylation profile.

Plant cell cultures as a system for the production of recombinant proteins, however, have several drawbacks, the main one of which is associated with a low yield of recombinant protein, which rarely exceeds 100 μg/kg of fresh weight, despite significant efforts to optimize protein expression and stability [6]. In whole plants, the problem of low yield of the target protein can be overcome by transient expression. Transient expression systems have surpassed the challenge of rapid and high-yield expression of recombinant proteins in plants, allowing for gram-sized quantities to be attained in as little as 5 days [7,8]. However, so far only a few examples of the use of a transient expression in suspension cell cultures are known in the literature [9,10]. One of the reasons for the low yield of recombinant protein is the insertion of the transgene into a random genome region, which leads to different levels of expression due to the positional effects and different numbers of transgene copies. The transgene can integrate into the chromatin region with varying degrees of compaction: into the region of active euchromatin or inactive heterochromatin, as well as in close proximity to native regulatory elements. What is more, epigenetic modifications such as methylation can also reduce transgene expression.

The problems of random integration can be overcome with the use of site-specific endonucleases. Such endonucleases, the most well-known of which is the Cas9 endonuclease, induce double-stranded breaks (DSB) in the target region of the genome, which are repaired by one of the two innate DNA repair systems: the non-homologous end-joining (NHEJ) pathway and the homology-directed repair (HDR) pathway [11,12,13]. If, during homologous repair, a donor template is provided that carries a sequence homologous to the site of a DSB or carries a whole additional gene, then the result of such repair can be a targeted gene change or a targeted insertion of an additional gene into a given region of the genome, the so-called knock-in [14,15,16]. Insertion of the target gene in the transcriptionally active region of the genome may provide it with a high level of expression. The possibility of creating “acceptor lines” in which reporter genes are inserted into regions well characterized for expression and then replaced with target genes using zinc finger nucleases is discussed in the paper of Schiermeyer et al. [17].

Using a donor format can influence the rate of HDR and editing outcomes. Donor templates can be delivered as dsDNA, such as PCR products, linearized or non-linearized plasmids, or ssDNA. The literature provides examples of the use of all types of templates for knock-in; however, ssDNA templates showed their low efficiency in plants, and linear DNA was usually delivered together with circular DNA, so it is difficult to assess the effect of template composition [18,19]. Circular DNA is most often used as a template for knock-in, and flanking the repair template with target sites and the release of linear repair template has been shown to provide a 2- to-5-fold improvement of targeted gene insertion in human culture cells in comparison with circular plasmid DNA [20,21]. In our study, we decided to test three types of templates for knock-in-target genes surrounded by homology arms, target genes surrounded by homology arms and flanked by the sequences recognized by Cas9, and target genes flanked only by the sequences recognized by Cas9 without homology arms.

Our study was aimed to test the possibility of integrating target genes into evolutionarily adapted regions of genes, the expression of which provides vital functions of the plant cell (regions of housekeeping genes). It should be emphasized that site-specific insertion of target genes into the genome of a plant cell is a very difficult task [15]. Moreover, when choosing such regions, the researcher can fail completely, since the introduction of foreign DNA into the regions of housekeeping genes can disrupt the coordinated expression of its own genes. In this regard, it is of interest to not only assess the possibility of stable expression of the target gene delivered to such a region but also demonstrate the features of expression of other genes in this region, as well as to evaluate the yield of the recombinant target protein.

Deltaferon (dIFN) encoded by the *dIFN* gene was chosen as the target recombinant protein. Deltaferon is a recombinant analog of human gamma-interferon, in which Arg-129 is replaced by Gly and Lys-130 is replaced by Ser and 10 C-terminal amino acid residues are deleted. Deltaferon is biologically active and more resistant to proteolysis than natural interferon gamma and has the same phagocytosis-stimulating properties as human IFN-gamma but reduced antiviral activity. According to its biological properties, it can be considered a component of medicines intended for the treatment of severe viral, immune, and oncological diseases [22,23]. For the targeted insertion of the dIFN gene, we selected the region of the histone H3.3 gene *HTR5* (At4g40040). Histone genes are the most important housekeeping genes since they provide the compaction of chromatin necessary for all eukaryotes, take part in the epigenetic regulation of gene expression, and are located in areas of actively transcribed chromatin. In plants, the most well-studied are the genes of histone H3 [24]. The haploid genome of *A. thaliana* contains 15 histone H3 genes, including five H3.1 genes, three H3.3 genes, and five H3.3-like genes. Histone H3.1 genes are expressed only in the S phase of the cell cycle and histone H3.3 genes are constitutively expressed during the entire interphase. Two of the three H3.3 genes *HTR5* and *HTR8* (At4g40040 and At5g10980) exhibited replication-independent expression in suspension cells [25]; among them the *HTR5* gene region looked more convenient for cloning.

Here, we describe the creation of three types of genetic constructs for the delivery of the target gene (*dIFN*) to the region of the *HTR5* gene, aimed at optimizing the frequency of knock-ins, receiving of transgenic cell suspension lines with the help of biolistic transformation, and a comparative analysis of the accumulation of the target recombinant protein (*dIFN*) in monoclonal cell lines with knock-in and cell lines with random insertions of the target gene. In addition, we evaluate the transcriptional activity of the *HTR5* gene and the adjacent gene encoding the 12 KDa subunit of microsomal signal peptidase in the *A. thaliana* monoclonal cell lines created. In this work, for the first time, the creation of plant cell suspension culture lines with a site-specific integration of the target gene obtained using CRISPR/Cas9 is described.

## 2. Materials and Methods

The fast-growing cell line of *A. thaliana* (L.) Heynh (Columbia ecotype, Col-0 inbred line) was used as the initial plant material. This cell line deposited in the All-Russian collection of higher plant cell cultures (ARCCCHP, http://www.ippras.ru/cfc/alccmp/, accessed on 16 August 2021) under No. 85 and designated as NFC-0 [26] was kindly provided by Ph.D. Nosov A.V. (Timiryazev Institute of Plant Physiology RAS, Moscow, Russia). The cell culture was maintained in vitro on SH medium [27] with the addition of phytohormones (1 mg/L 2,4-D and 0.1 mg/L kinetin).

### 2.1. Plasmids Carrying Cas9 and Guide RNA

The plasmids pBlu/gRNA (#59188) for an intermediate cloning step and Cas9 MDC123 (#59184) with the Cas9 endonuclease gene under the control of the 2 × 35S CaMV promoter optimized for expression in Glycine max cells were donated by R. Stupar [28] from the Addgene repository. The pBlu/gRNA plasmid carrying an sgRNA cassette under the control of the *A. thaliana* U6 promoter was used as an intermediate vector for inserting selected spacer regions into the sgRNA sequence.

### 2.2. The Sources of Elements of Genetic Constructs for Knock-In

Nucleotide sequences for creating genetic constructs were obtained by PCR using appropriate oligonucleotides and templates. The genomic DNA of *A. thaliana* was used as a template for the amplification of sequences flanking the integration site of the target gene. The signal peptide sequence directing the synthesized protein to the apoplast was amplified on a *Daucus carota* genomic DNA template. The plasmid pGEX4T-1 was the source of the *GST* (glutathione S-transferase) gene encoding the tag for affinity purification of the target protein. For the amplification of the *nptII* gene sequences (neomycin phosphotransferase II) and the CaMV35S promoter of the cauliflower mosaic virus, the pBi121 plasmid was used as a template. The *dIFN* (deltaferon) gene, a recombinant analog of human interferon gamma for the plant cells expression, was amplified from the pIFN-γ-trp2-Δ plasmid [29].

### 2.3. Selection of Guide RNA

The region of the histone H3.3 gene *HTR5* was chosen as the region for the integration of the target gene. The CRISPOR software (http://crispor.tefor.net/crispor.py, accessed on 16 August 2021) was used to select the guide RNA for knock-in into the region of the *HTR5* gene. The sequence for knock-in was located between the locus of histone H3.3 gene *HTR5* (At4g40040) and the adjacent gene encoding the 12 kDa subunit of microsomal signal peptidase (At4g40042), preceding the coding region of the *HTR5* gene.

### 2.4. Creation of a Cas9 (H3.3) Genetic Construct for Introducing Double-Strand Breaks into Target Regions

The selected sequences that determine the specificity of the targeting sgRNA were transferred into the Cas9 MDC123 plasmid by means of the pBlu/gRNA intermediate plasmid using the corresponding oligonucleotides (Supplementary Materials Table S1): for the region of the *HTR5* gene–1_gRNA_H Up and 1_gRNA_H Lo. The first stage of the construct assembly consisted of hybridization of the selected phosphorylated oligonucleotides and their subsequent integration into the pBlu/gRNA plasmid treated with the BstV2I restriction enzyme (Sibenzyme, Novosibirsk, Russia). To confirm the insertion of the target sequence, the DNA of the resulting clones was sequenced using a standard T3 primer (Supplementary Materials, Table S1). The second stage of the construct assembly consisted in the transfer of the obtained sgRNA carrying the targeting sequence into the plasmid Cas9 MDC123. The insertion was performed at the *Eco*RI endonuclease restriction site.

### 2.5. Creation of the Genetic Constructs pIFN (H3.3).1, pIFN (H3.3).2 and pIFN (H3.3).3, Carrying a Template for Homologous Recombination

**Figure 1:**
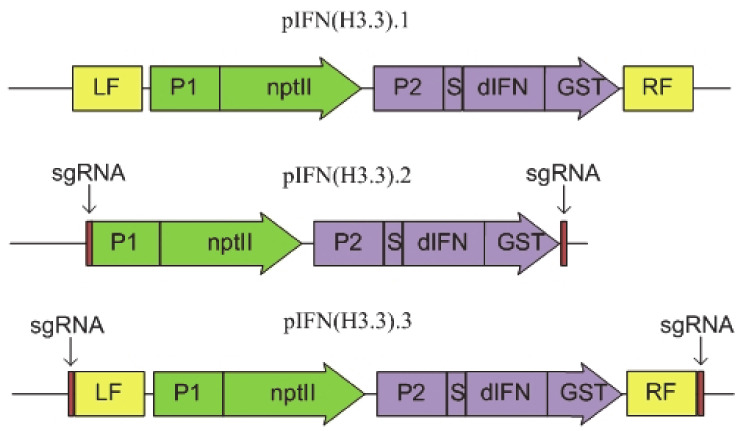
Schemes of genetic constructs for the delivery of the *dIFN* gene to the region of the *HTR5*.

To deliver the desired gene *dIFN*, encoding a human γ-interferon, and the selective *nptII* gene, conferring kanamycin resistance to transformed cells, into the location of histone H3.3 gene, three types of genetic constructs, pIFN (H3.3).1, pIFN (H3.3).2 and pIFN (H3.3).3, were designed. Schemes of their structure are presented in Figure 1. To optimize the integration frequency of the latter two constructs into the target region, they were flanked by the sequences recognized by sgRNA, which ensured their excision from a plasmid in the cell as a linear structure.

To obtain the target deltaferon gene, with a signal peptide and an attached *GST* gene, primers Up_sig.BglII(62), Lo_dIFN(60), ifn + Up_GST(60), and Lo_GST(GEX) stopA65I(66) were used (all sequences of the primers used are given in Supplementary Materials Table S1). The first two primers were used to generate the *dIFN* gene with sequence coding of the signal peptide. In order for the chimeric *dIFN-GST* gene to be translated entirely, the stop codon was removed from the deltaferon gene in the resulting DNA fragment. Primers ifn + Up_GST and Lo_GST(GEX)stopA65I(66) were used to obtain the *GST* gene sequence. Then primers Up_sig.BglII(62), Lo_GST(GEX) stopA65I(66) and both DNA fragments obtained were used in PCR to obtain the target DNA fragment signal--his6-dIFN-GST.

To obtain the three final constructs, three intermediate plasmids were created. All intermediate plasmids were obtained on the basis of the pUC19 vector.

The intermediate plasmid pInt_var.1 carries sequences from the *A. thaliana* genome flanking the insertion region and a kanamycin resistance gene with a promoter and terminator. To obtain pInt_var.1, three DNA fragments were preliminarily obtained. The first two fragments, LF and RF (flank sequences), were synthesized by two sequential PCRs on a template of *A. thaliana* genomic DNA with primers: Up_L_HIII and Lo_L_Acc_Sfr (LF) and Up_R_Sfr_Acc and Lo_R_Sal (RF). The third fragment (*nptII* gene with p35S promoter) was obtained by PCR on the pBi121 plasmid template with primers Lo_A-B_pBI(58) and Up_Xho-pBI(60). Using the LF and RF fragments, as well as the primers Up_Xho-pBI(60), Up_L_HIII, and Lo_R_Sal, an LF-RF DNA fragment containing recognition sites for the Acc65I, Sfr274I restrictases between LF and RF was obtained, which was then inserted into pUC19. A DNA fragment carrying the *nptII* gene was cloned into pUC-LF-RF at the Sfr274I/Acc65I restriction enzyme recognition sites.

The intermediate plasmid pInt_var.2, instead of the flanking DNA of Arabidopsis, carries the Cas9 endonuclease recognition sites identical to the site of the double-stranded break in front of the *A. thaliana HTR5* gene as well as the *nptII* gene with a promoter and terminator. To obtain the nptII fragment, the primers Up_Sal_gRNA-pBI(60) and Lo_A-B_pBI(58) were used instead of the primers Lo_A-B_pBI(58) and Up_Xho-pBI(60). The resulting DNA fragment was cloned into pUC19 at the SalI and Acc65I restriction enzyme recognition sites. The target signal-his6-dIFN-GST fragment was obtained using the Lo_Acc_gRNA_GST(60) primer instead of Lo_GST(GEX)stopA65I(66).

The intermediate plasmid pInt_var.3 carries sequences from the Arabidopsis genome flanking the insertion region, recognition sites for the Cas9 endonuclease that are identical to the double-strand break site upstream of the *A. thaliana HTR5* gene, and the *nptII* gene with a promoter and terminator. Plasmid pInt_var.3 was obtained similarly to plasmid pInt_var.1, but instead of primers Up_L_HIII and Lo_R_Sal, primers Up_L_HIII_gRNA and Lo_R_Sal_gRNA were used for cloning.

The resulting target DNA fragment signal-his6-dIFN-GST was used for cloning within the intermediate plasmids pInt_var.1, pInt_var.2, and pInt_var.3 at the recognition sites of the BglII/Acc65I restriction enzymes to form three target plasmids, pIFN (H3.3).1, pIFN (H3.3).2 and pIFN (H3.3).3.

### 2.6. Biolistic Transformation of A. thaliana Cells and Obtaining Transgenic Suspension Cultures

Delivery of the target *dIFN* gene to the region of the *HTR5* gene of *A. thaliana* was carried out using biolistic transformation with the immobilization of plasmids pIFN (H3.3).1, pIFN (H3.3).2 or pIFN (H3.3).3 together with the plasmid Cas9H33 on the gold particles. In total, 10 biolistic transformations were performed for each construct.

For the transformation by the biolistic method, 1 mL of cell suspension was applied to the surface of the SH medium [27] with the addition of phytohormones (1 mg/L 2,4-D and 0.1 mg/L kinetin), distributing it evenly, and grown in the dark within 3 days for the formation of a callus cell layer. Calluses were transformed using a PDS1000/He system (Bio-Rad, Hercules, CA, USA), using the following biolistic parameters: gold particle size—0.6 µm; membrane rupture pressure—1100 psi; vacuum pressure in the chamber—27 inches Hg; distance to the explant—6 cm. Immobilization onto gold particles of an equimolar mixture of two plasmids, one of which included the target gene, and the second with the Cas9H3.3, was carried out according to the method of the gene gun manufacturer. For each transformation, 6 Petri dishes with prepared calluses were used; each callus was fired on twice.

Three days after the biolistic transformations, the calluses were transferred to a selective SH medium of the same composition supplied with kanamycin at a concentration of 100 mg/L and cultured in a light with an intensity of 20,000 lux at a photoperiod of 18/6 h (day/night). Transfer of the calluses to fresh media of the same composition were carried out weekly. The resulting antibiotic resistant calluses were used to obtain cell suspensions. The suspensions were cultured in the dark, using an orbital shaker at 160× *g*. Furthermore, monoclones were obtained from these cell cultures. For this, on the 4th day of cultivation, cell suspensions diluted with the culture medium were seeded on Petri dishes with SH medium supplied with the antibiotic kanamycin (100 mg/L), and microcalluses were grown from individual cells/cell aggregates.

### 2.7. Identification of Monoclonal Cell Lines with the Target Insertion of Genes (Knock-Ins)

The detection of knock-ins was carried out using PCR and sequencing DNA of kanamycin-resistant calluses. Genomic DNA was isolated from the calluses using CTAB buffer according to the Allen protocol [30]. PCR analysis and sequencing (at ZAO Evrogen, Moscow, Russia) were performed using the appropriate primers (Supplementary Materials Table S1). The monoclonal cell lines were tested using primers for the selective *nptII* gene, for the target *dIFN* gene, and for the event of target insertion—when one primer is located in the target region of plant DNA, and the other is located in the transgenic construct.

### 2.8. Identification of Monoclonal Cell Lines with Random Insertion of Transgenes

The control group with random insertion of transgenes included cell lines identified by resistance to the antibiotic kanamycin, in which the presence of the marker *nptII* gene and the target *dIFN* gene was confirmed by PCR analysis, but insertion into the specified region of the genome was not confirmed. The detection of insertions of target genes was carried out using PCR and DNA sequencing of calluses resistant to selective agents. Genomic DNA was isolated using CTAB buffer according to the Allen protocol [30]. PCR analysis and DNA sequencing (at ZAO Evrogen, Moscow, Russia) were performed using the appropriate primers (Supplementary Materials, Table S1). The monoclonal cell lines with random insertion of transgenes were tested using primers for the selective *nptII* gene, for the target *dIFN* gene, and for the event of target insertion—when one primer is located in the target region of plant DNA, and the other is located in the transgenic construct.

### 2.9. Analysis of A. thaliana Gene Expression Surrounding the Target Site

Total RNA from the cell biomass of each monoclonal line was isolated using the ExtractRNA reagent (Evrogen, Russia), RNA was treated with DNase I (Thermo Scientific, Riga, Latvia), and 4 µg RNA was used to obtain cDNA (Thermo Scientific RevertAid First Strand cDNA Synthesis Kit, Latvia). Expression analysis was performed using real-time PCR on a CFX96 amplifier (Bio-Rad, CA, USA). The reaction was carried out in 20 μL of a reaction mixture of the following composition: 15 ng of the studied cDNA, 50 mM Tris-SO_4_, pH 9.0, 30 mM KCl, 10 mM ammonium sulfate, 0.01% Tween 20, 3 mM MgCl_2_, 0.2 mM of each deoxyribonucleoside triphosphate, 0.4 μM of each primer, 0.4 μM of each probe, and 0.1 u.a./μL Hot Start DNA polymerase (Biosan, Novosibirsk, Russia). To create the probes, we used pairs of fluorophore-quencher: FAM-BHQ1 and Cy5-BHQ2. Real-time PCR was carried out in the multiplex version. The level of target gene expression was investigated relative to the *HTR5* gene (primers and probe HIS3.3_f, HIS3.3_r, HIS3.3_p), the gene encoding the 12 kDa subunit of microsomal signal peptidase (primers and probe VAP27-1_f VAP27 -1_r VAP27-1_p), as well as *A. thaliana*’s own *PARP2* gene (primers and probe PARP2_f, PARP2_f, PARP2_r). The structures of primers and probes are shown in Supplementary Materials (Table S1). Amplification was carried out according to the following scheme: 3 min at 95 °C, then 40 cycles with fluorescence detection at the annealing stage: 10 s at 95 °C, 20 s at 61 °C. The total RNA of the initial non-transgenic cell line served as a control in this experiment. All measurements were performed in three biological replicates.

Data processing was carried out using Bio-Rad CFX Manager 3.1 software. Normalized expression was calculated using the formula:

(1) $$\frac{\mathrm{dIFN}\;\mathrm{expression}}{\mathrm{PARP}2\;\mathrm{expression}}={\left(1+E\right)}^{Ct_{\mathrm{dIFNTest}}-Ct_{\mathrm{PARP}2\mathrm{Test}}+Ct_{\mathrm{PARP}2\mathrm{Control}}-Ct_{\mathrm{dIFNControl}}}$$

where *E* is the PCR efficiency and Ct the threshold fluorescence cycle in the test and control samples, respectively.

### 2.10. ELISA of Target Protein (dIFN) in Monoclonal Cell Lines

For ELISA, we used protein extracts obtained from a series of *A. thaliana* monoclonal cell lines with target insertions of the *dIFN* gene in the region of the *HTR5* gene and 7 cell lines with insertions of the studied gene into the random regions of the genome. Protein extracts of *A. thaliana* non-transgenic cell culture served as a negative control. Protein extract of a non-transgenic cell culture of *A. thaliana* supplied with 1 μg/μL of dIFN, which we had previously synthesized in *E. coli*, was used as a positive control. dIFN in *E. coli* cells was synthesized on the same matrix that was used in genetic constructs for the production of knock-ins in plant cells.

To extract proteins, 40 mL of a 7-day cell suspension culture was centrifuged for 10 min at 12,000× *g* (Allegra X-30R, Beckman Coulter, Loveland, CO, USA). The cell precipitate was triturated in liquid nitrogen and a 0.7 g sample was transferred to a 2 mL tube with the addition of 1400 μL of PBS buffer (137 mM NaCl, 2.7 mM KCl, 10 mM Na_2_HPO4, and 1.8 mM KH_2_PO_4_ with 8 M urea). Then, the extracts were homogenized on Vibra-Cell™ Ultrasonic Liquid Processors VCX130 (USA) according to the program: 5 cycles, amplitude 45%, 10 s on, 15 s off. The extracts were centrifuged for 10 min at 14,000× *g* (Eppendorf, Hamburg, Germany) and the supernatant was collected in a separate tube.

The protein extract (100 μL) in four replicates was introduced into the wells of a 96-well MICROLON^®^ plate (Greiner Bio-One, Frickenhausen, Germany). The sorption on the plate was carried out overnight at a temperature of 10 °C. After sorption, the wells were washed with 250 μL of PBST (PBS buffer with the addition of 0.1% Tween 20) and then 250 μL of 0.5% fat-free dry milk in PBS was added to each well and incubated for 30 min at room temperature with shaking, after which the wells were rinsed again with 250 μL PBST. The remaining liquid was removed with a water-jet pump. In total, 100 μL of primary antibodies (anti-interferon gamma (human) antibodies IMS01-145-319, Agrisera, Sweden) diluted in PBS buffer (1:5000) was added to each well and the plates were incubated with shaking for 1 h at room temperature. After washing three times with 300 μL PBST, 100 μL of secondary antibodies (Goat Anti-Chicken IgY (H&L), HRP conjugated, AS09 603, Agrisera, Sweden) diluted in PBS (1:10000) was added to each well and incubated with shaking for 1 h. After washing the wells three times with 300 μL PBST, 100 μL TMB (3,3′,5,5′-tetramethylbenzidine; Abcam, UK) was added to each well and incubated with shaking for 30 min in the dark at room temperature. The reaction was stopped by adding 100 μL of 0.1 M HCl. Within 15 min after stopping the reaction, the optical density was measured on a Victor X3 2030 device (PerkinElmer, Akron, OH, USA) at a wavelength of 450 nm. All measurements were carried out in three biological replicates. The optical density (OD) value of the negative control was subtracted from the OD of all experimental values and the positive control. The accumulation of the target dIFN protein in the analyzed cell lines was estimated as a percentage of the TSP. The concentration of TSP was determined by the Bradford method [31] using BSA solution in the PBS buffer as standards. The measurements were carried out in three biological replicates. The ELISA results were statistically processed using the Statistica package.

## 3. Results

### 3.1. Delivery of the Genetic Constructs to the Selected Target Region of the A. thaliana Genome

The results of the efficiency of *dIFN* and *nptII* gene delivery to the target region of the *HTR5* gene using three types of genetic constructs are presented in Table 1.

As can be seen in Table 1, three genetic constructs differed in the delivery efficiency of target genes in a target region. The largest number of knock-ins in the target site, six lines, were obtained using the pIFN(H3.3).3 genetic construct, while only three lines were obtained using the pIFN(H3.3).2 construct. The pIFN(H3.3).1 construct turned out to be ineffective since none of the 10 biolistic transformations did reveal a single integration event of the target gene in the target region. The results obtained indicate that the addition of Cas9 nuclease recognition sites intended for excision of the target DNA from the carrier plasmid in the plant cell to the flanking DNA identical to the insertion site increases the likelihood of delivery of the target genes to the target region.

After biolistic transformation, calli cultivated on a culture medium with kanamycin represent a heterogeneous mass of cells, including both cells with knock-in events in the target region and cells with the integration of target genes into random regions of the genome. To obtain monoclonal cell lines, we used one part of the kanamycin-resistant callus for analysis for the presence of knock-ins, while the second part was transferred to a fresh medium. It is quite obvious that such a procedure for selecting cells with knock-ins may be associated with their loss. As can be seen from the data in Table 1, only two lines carrying the knock-in with the construct pIFN(H3.3).2 and two lines carrying the knock-in with the construct pIFN(H3.3).3 served as progenitors for the creation of monoclonal cell lines. The rest of the lines were lost during cloning and transplanting.

**Figure 2:**
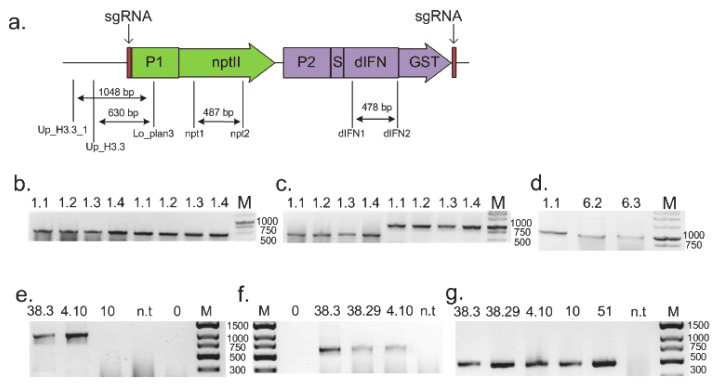
PCR analysis of the obtained cell lines, confirming the integration of the target gene into the target region of the *HTR5* gene. (**a**) The location of primers for testing using the pIFN(H3.3).2 construct as an example: P1—P-NOS-promoter of *A. tumefaciens* nopaline synthase gene; *nptII*—gene of neomycin phosphotransferase II, which provides plant cell resistance to kanamycin; P2—CaMV35S promoter of the cauliflower mosaic virus; S—DNA sequence encoding the leader signal of the carrot extensin gene, which ensures the transport of deltaferon to the apoplast; dIFN—DNA sequence encoding the target protein deltaferon; GST—DNA sequence encoding the GST tag; sgRNA—20 bp Cas9 endonuclease recognition sites, identical to the recognition site in the intergenic region upstream of the *A. thaliana HTR5* gene, for excision of the construct from the plasmid in the cell. At the bottom, the names of the oligonucleotides used and the size of the PCR fragments obtained with them are indicated. (**b**–**d**) Electrophoresis of PCR products of cell lines produced with the pIFN(H3.3).2 construct. (**b**) PCR with primers for the *nptII* gene (lanes 1–4; the size of the expected fragment is 487 bp) and for the *dIFN* gene (lanes 5–8; the size of the expected fragment 478 bp). (**c**) PCR with primers for the target insertion into the region of the *HTR5* gene (lanes 1–4—with primers Up_H3.3/Lo_plan3 (630 bp) and lanes 5–8—with primers Up_H3.3.1/Lo_plan3 (1048 bp)). (**d**) PCR with primers for the target insertion into the region of the *HTR5* gene with primers Up_H3.3.1/Lo_plan3 (1048 bp). Cell line numbers are indicated above the gel lanes. Cell lines 1.1–1.4 and 6.2–6.3 are knock-ins. Lane M is a 1 kb DNA fragment length marker (SibEnzyme, Novosibirsk, Russia). (**e**–**g**) Electrophoresis of PCR products of cell lines produced with pIFN(H3.3).3 construct. (**e**) PCR with primers Up_H3.3/Lo_plan3 (1048 bp) for the target insertion into the region of the *HTR5* gene. (**f**) PCR with primers Up_H3.3/Lo_plan3 (630 bp) for the target insertion into the region of the *HTR5* gene. (**g**) PCR with primers npt1 and npt2 for the *nptII* gene (487 bp). Cell line numbers are indicated above the gel lanes. Cell lines 38.3, 38.29 and 4.10 are knock-ins. Cell lines 10 and 51 have random insertion of the transgene constructs pIFN(H3.3).2 and pIFN(H3.3).3 correspondingly. Lane n.t. is non-transgenic *A. thaliana* (negative control 1). Lane 0 is non-template DNA (negative control 2). Lane M is a DNA fragment length marker (GeneRuler Express DNA Ladder; Thermo Fisher, Lenexa, KS, USA).

To confirm the integration of the target gene into the target region of the *HTR5* gene, PCR was used with primers, one of which was located inside the transferred construct and the other on the plant DNA region outside the flanking sequence present in the construct (Lo_plan3 and Up_H3.3_1, Supplementary Materials Table S1). Additionally, control PCR was performed with primers located inside the construct (Lo_plan3 and Up_H3.3, dIFN1 and dIFN2; Supplementary Materials Table S1, Figure 2a). The presence of fragments of the expected size indicated that in all four cell lines, the integration of the target genes occurred in the given target region (Figure 2b–g).

Alignment (Supplementary Materials Figure S1) of the nucleotide sequence read during sequencing of PCR fragments obtained with primers Lo_plan3 and Up_H3.3_1 and genomic DNA of the obtained knock-ins showed identity with *A. thaliana* histone superfamily protein (AT4G40040) when sequenced from one side and identity with vector DNA (namely, with the promoter of the *nptII* gene) when read from the other side. During the alignment, slight rearrangements were noted at the junction of the plant and vector DNA, especially in line 1 obtained with the construct pIFN(H3.3).2, which is integrated into the genome by the mechanism of NHEJ. The results of sequencing a specific PCR fragment confirmed the targeted insertion of the genetic construct into the target region of the histone gene *H3.3*. As a result of the biolistic transformation of two types of genetic constructs, 4 cell lines with target gene knock-ins in the target region of the *HTR5* gene were obtained, which served as progenitors for the creation of monoclonal cell lines.

### 3.2. Establishment of A. thaliana Monoclonal Cell Lines with Target Gene Knock-Ins in the Region of the HTR5 Gene and Monoclonal Cell Lines with Random Insertions into the Genome

Six monoclones were selected from two lines with the integration of the target gene into the *HTR5* gene region obtained using the pIFN(H3.3).2 construct: four for line 1 (1.1–1.4) and two for line 6 (6.2 and 6.3). Nine monoclones were selected from two lines with knock-in obtained using the pIFN(H3.3).3 construct: four for line 38 (38.3; 38.8; 38.22 and 38.29) and five for line 4 (4.10; 4.15; 4.25; 4.27 and 4.28).

For a comparative analysis of the expression of the target gene delivered to the target region and assessment of the level of accumulation of the target protein in the monoclonal cell lines, seven monoclonal cell lines with insertions of the target gene into random regions of the genome were selected. Monoclones 41 and 51 were selected from cell lines obtained using the pIFN(H3.3).3 construct. Five monoclones were selected from cell lines obtained with a different construct pIFN(H3.3).2 (2.1; 2.2; 8.1; 10.1 and 10.4).

### 3.3. Analysis of dIFN Gene Expression and Accumulation of the dIFN Protein in Monoclonal Cell Lines with a Knock-In in the Target Region of the Genome and in Monoclonal Cell Lines with Random Insertions into the Genome

**Figure 3:**
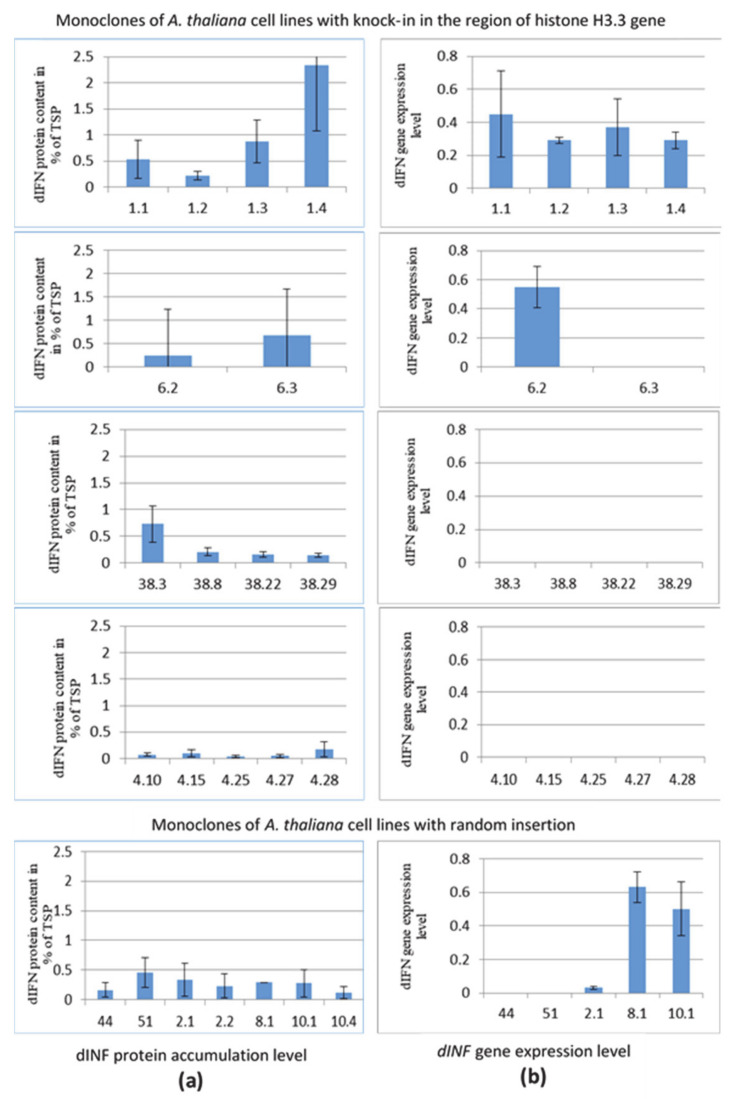
The level of *dIFN* gene expression and dIFN protein accumulation in monoclones of *A. thaliana* transgenic cell lines with knock-in in the region of the *HTR5* gene and in monoclones of *A. thaliana* transgenic cell lines with random insertion. (**a**) dIFN protein accumulation level, data normalized to the content of TSP, the sample size was 100 μL of protein extract; (**b**) analysis of the level of *dIFN* gene expression by quantitative RT-PCR; data were normalized to the *PARP2* gene; the sample size was 15 ng of the cDNA. The standard error of the mean is shown. The numbers of the corresponding cell lines are indicated under the bars.

The accumulation of the target dIFN protein in the analyzed cell lines, estimated as a percentage of the total soluble protein (TSP), was investigated by ELISA. The results of ELISA are shown in Figure 3A. Among the four cell lines with the target gene delivered to the region of the *HTR5* gene, a consistently high level of accumulation of the dIFN protein was not observed. As can be seen in the figure, all four lines differed in the level of accumulation of the target protein. Moreover, in each line, the dIFN content varied between individual monoclones. The highest level of accumulation of the dIFN protein was found in monoclones of line 1; in the case of monoclone 1.4, it reached more than 2% of the TSP. Statistical analysis of the data obtained by the Kruskal–Wallis test revealed significant differences between the monoclones H(3, N = 15) = 9802, *p* = 0.0203. Analysis of the data obtained using Dunn’s test, taking into account multiple comparisons, revealed significant differences only between lines 4 and 1 (Q = 2,950, k = 4, α = 0.05, with Qst = 2639).

Based on the data of T. Okada et al. [25], who revealed a high level of expression of the *HTR5* gene in *A. thaliana* cell cultures, this region was chosen as the target region for the delivery of the *dIFN* gene. In this regard, it was of interest to determine the amount of mRNA of the target gene placed in the region with a high transcriptional activity using the real-time PCR method. Figure 3b shows the results of the analysis of *dIFN* gene expression normalized to the expression of *A. thaliana*’s own *PARP2* gene. It was found that the mRNA of the *dIFN* gene was not detected in all lines. The highest expression level was observed in monoclones of line 1, as well as in lines with random insertion. In monoclones of lines 38 and 4, expression of the *dIFN* gene was not detected, which correlated with the low level of protein in these lines.

For a comparative analysis of the level of accumulation of the target protein integrated into the transcriptionally active region, and the protein delivered to random regions of the genome, the mean values of protein accumulation in monoclones of one cell line were compared. The mean content of dIFN protein in the group of monoclones of line 4 was 0.085%, line 38–0.310%, line 1–0.990% and line 6–0.459%. The mean dIFN content for lines with random insertion was 0.160% (line 44), 0.454% (line 51), 0.282% (line 2), 0.291% (line 8), and 0.194% (line 10). As a result of comparing the mean protein content in the lines with knock-in and random insertion, no significant differences were found by Student’s test, *t* = 1.0345, df = 7 tst (df = 7) = 2.365.

### 3.4. Analysis of the Expression Level of A. thaliana’s Own Genes Surrounding the Knock-In Site

**Figure 4:**
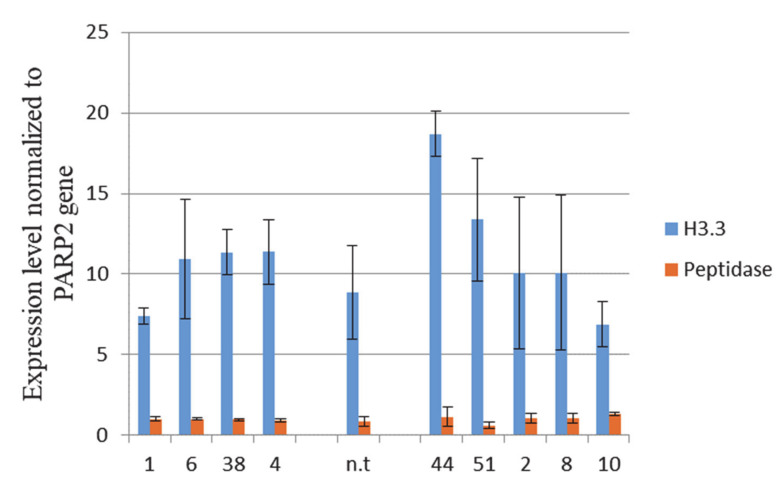
Expression level of the *HTR5* gene and the gene encoding the 12 kDa subunit of microsomal signal peptidase in *A. thaliana* cell lines with knock-ins in the region of *HTR5* gene (line numbers 1, 6, 38, and 4) in the nontransgene line (nt) and in transgenic lines with random insertion (line numbers 44, 51, 2, 8, and 10). Quantitative RT-PCR data normalized to the *PARP2* gene are shown. The standard error of the mean is shown.

Since the site of insertion of the target construct is located in the promoter region of two *A. thaliana* genes the *HTR5* gene and the gene encoding the 12 kDa subunit of microsomal signal peptidase, it was of interest to check whether the insertion of the target genes affected their expression. Figure 4 shows the results of evaluating the expression of the *HTR5* gene and the gene encoding the 12 kDa subunit of microsomal signal peptidase in *A. thaliana* monoclonal cell lines with a knock-in in the region of *HTR5* (line numbers 1, 6, 38, and 4). A cell line (n.t.) obtained from the non-transgenic callus of *A. thaliana* (control 1) and monoclonal lines with random insertion (line numbers 44, 51, 2, 8, and 10; control 2) were used as controls for comparison. For lines 1, 6, 38, and 4, the data on the expression of the studied genes were pooled, since no significant differences were found between the monoclones for each of these lines. Figure 4 shows the mean values for the expression of the *HTR5* genes and 12 kDa subunit of the microsomal signal peptidase gene obtained for all monoclones in each line.

As we can see in the figure presented, both the studied genes significantly differed from each other in terms of expression level. Compared to the *HTR5*gene, the microsomal signal peptidase gene was much weaker expressed in all the analyzed cell lines. Comparative analysis using the nonparametric Kruskal–Wallis test did not reveal significant differences in the expression level of the *HTR5* gene and the microsomal signal peptidase gene between the group of lines with site-specific insertion and control groups (lines with random insertion and a non-transgenic line). Thus, it becomes obvious that the level of expression of *A. thaliana*’s own genes little, if not, differs in lines with knock-in and with random insertion of the construct, which suggests that the insertion of the target construct into the studied target region of the *HTR5* gene did not affect the expression of the studied plant genes.

## 4. Discussion

Despite the attractiveness of using higher plant cell cultures for the commercial production of biopharmaceuticals, the yield of recombinant protein in this expression system is still low compared to animal cell cultures. Typically, the yield of the recombinant protein is about 1% of the TSP [32,33]. In whole plants, the problem of low yield can be solved by transient expression or expression of the target gene in chloroplasts [1,8]. To increase the biosynthesis of recombinant proteins in cell suspension culture, researchers use various methods, including optimizing the expression of target genes due to regulatory elements in expression cassettes, reducing the degradation of target proteins, optimizing the conditions for cultivating plant cells, etc. [34,35]. In general, the problem of increasing the biosynthesis of recombinant proteins is solved through the search (screening) of the most “successful” events of insertion of the target gene into the plant genome and selection of “elite” highly productive cells. Then, based on “elite” cells, monoclonal suspension cell cultures are formed [36]. After successful delivery of the target gene into the genome, the plant cell culture is a heterogeneous mass of genetically and epigenetically different cells with different expression levels, the number of transgenic copies, and different insertion sites, all of which significantly affect their productivity. Only a small proportion of cells from the original transformants remain capable of producing the recombinant protein with high efficiency.

The observed variability in the ability to biosynthesize and accumulate recombinant proteins in plant cells is largely determined by the organization of the transgene insertion region. The optimally high level of target gene expression, planned by the researchers during the design of the genetic construct, may not be realized when the transgene enters the transcriptionally inactive regions of the genome. Thus, the identification of transcriptionally active regions of the genome and directed integration of target genes into these regions opens up new prospects for researchers to increase the synthetic capabilities of plant cells for the production of recombinant proteins.

The rapid development of genomic editing methods using CRISPR/Cas9 opens up the opportunity for researchers to deliver target genes to constitutively transcribed regions of the genome and to obtain highly productive lines producing recombinant proteins. Plant cell housekeeping genes that are actively expressed during the entire interphase of the cell cycle can be potential target sites for the integration of target genes. We tried to implement this approach in this study and evaluate the possibilities of increasing the accumulation of recombinant protein in the case of integration of the target *dIFN* gene into the region of one of the housekeeping genes—the histone H3.3 gene *HTR5*.

Despite a significant increase in the number of works on editing the plant cell genome, the number of experimental works devoted to gene insertion using the knock-in method is still small [13,16]. The DNA DSB, which is formed as a result of the action of Cas9, initiates cellular repair mechanisms, thereby making the break site available for insertion of donor DNA. In plant cells, as in most eukaryotic cells, DSB are usually repaired through the NHEJ mechanism, which functions throughout the entire cell cycle, except for mitosis. The HDR pathway, which provides accurate sequencing, is only possible in the presence of a sister chromatid (homologous template) and can only occur at the end of the S and G2 phases of the cell cycle [14,15,37]. Thus, the predominance of the first mechanism over the second in plants poses very difficult tasks for researchers in the case of attempts to carry out genomic editing in the knock-in variant. Indeed, in plants, the frequency of knock-out obtained during the restoration of DSB by the NHEJ mechanism is 30–70%, and in some cases up to 100%, while the knock-in frequency is usually at a level of a part of a percent or a few percent [15,16].

As a rule, most of the genetic constructs intended for the delivery of transgenes to the target region using the CRISPR/Cas9 genomic editing technology include flanking DNA regions homologous to the insertion site [21,38,39,40,41]. To increase the efficiency of delivery of the target construct to a given region of the genome of *A. thaliana* plants [38] and *Zea mays* [21,41], a technique was used that allows the release of the target fragment in the cell in the form of a linear template from the circular plasmid DNA with the help of Cas9 nuclease. In experiments of Peterson and colleagues, direct comparison of T-DNA vectors with and without target sites flanking repair templates consistently demonstrated an approximately three-fold increase in targeted gene insertion frequency [21]. The use of this approach made it possible to deliver target genes to the target region with a site-specific insertion efficiency of 0.14% for *A. thaliana* and 4.7% for *Zea mays* [38,41]. The advancements in Agrobacterium-mediated maize transformation, combined with optimized vector design, enabled an approximately 100-fold improvement in the efficiency of targeted gene insertion [21]. Adding Cas9 nuclease recognition sites was effective not only in the case of flanking DNA homologous to the insertion site (pIFN(H3.3)3 construct) but also in the absence of such flanks (pIFN(H3.3)2 construct). Thus, in genomic editing of cell cultures in the knock-in variant, the presence of plant DNA homology arms alone is not enough for successful site-specific insertion of the target gene. When the pIFN(H3.3).1 genetic construct was used, as a result of 10 biolistic transformations, not a single event of *dIFN* gene delivery to the region of the *HTR5* gene was detected. At the same time, the inclusion of Cas9 nuclease recognition sites in the flanking regions in the pIFN(H3.3).3 construct made it possible to identify six events of transgene delivery to the target region with the same number of biolistic transformations performed. Although in the case of using the pIFN(H3.3)2 construct, only three desired events were identified, this approach can also be successfully used in the work. The absence of flanking DNA simplifies the process of creating a genetic construct associated with the addition of homology regions to the construct or, on the contrary, their replacement when the target site is changed. Thus, as the experiments have shown, the introduction of Cas9 recognition sites into the genetic construct, intended for its excision from the circular plasmid in the cell, seems to be a very effective approach for integrating transgenes into target regions of plant cell cultures. It seems more promising to us to use a template without flanking homology arms and carrying only the Cas9 recognition sites, since such a design, based on insertion by the NHEJ mechanism, excludes additional stages of construction and, with a fairly high frequency, can lead to the successful production of lines with knock-in.

It is worth emphasizing that an important problem that remains is the low efficiency of the selection of cells carrying knock-in since plant cells in suspension culture are prone to the formation of cell aggregates. Separating knock-in cells from cells in which the same construct is integrated into a random region of the genome is a laborious task. It is desirable to monitor the presence of knock-in regularly even among cells that have successfully passed selection and carry a confirmed knock-in, since it is known that cells that produce foreign proteins at a high level tend to be displaced from a heterogeneous culture [36,42]. To reduce the heterogeneity of the cell culture and obtain monoclonal lines with knock-ins, it is necessary to control the cell population for the presence of knock-in, since the heterogeneity of the culture also often leads to loss of cells carrying the transgene [43,44,45] or knock-in. We faced such a problem in our experiment—some of the cell lines with knock-ins were lost during their selection and creation of monoclonal cell lines based on them. Seven kanamycin-resistant cell lines, in which PCR with genomic DNA did not reveal fragments of the corresponding size, indicating the insertion of the transgene into the target region of the *HTR5* gene, formed the basis for the creation of monoclonal cell lines with random insertions.

The created series of monoclonal cell lines served as a convenient model for analyzing the expression features of the target *dIFN* gene integrated into the region of the *HTR5* gene, characterized by high transcriptional activity, and assessing the possibility of increasing the accumulation of the recombinant protein. Contrary to expectations, a high level of accumulation of the dIFN protein was found only among monoclones of line 1, which reached more than 2% of the TSP in the case of monoclone 1.4. In general, the accumulation of the target protein varied both between cell lines and between individual monoclones derived from this line. The region of the *HTR5* gene was used as a target for the delivery of the *dIFN* gene due to its high transcriptional activity in the cells of the *A. thaliana* suspension culture [15]; however, the results of RT-PCR did not reveal a direct correlation between protein accumulation and the amount of mRNA with which this protein is synthesized. Moreover, for lines 38 and 4, mRNA was not detected at all, but the protein was detected in monoclones.

The observed high variability in the level of accumulation of the dIFN protein in lines with knock-in can be possibly explained by the fact that during insertion, mutations occur in the regulatory region of the target genes that affect their expression. The contribution of somaclonal variability in the diversity between cell lines in target gene expression and protein accumulation is also not excluded. The variability in the expression of several genes integrated into the rice genome using recombinase is shown in the work [46]. Forsyth et al. [47] did not observe any variability in the expression of marker genes integrated into the promoter region of the potato *Ubi7* gene using TALEN. However, in this work, the target marker genes were inserted into the genome in such a way that they are placed under the control of the native promoter (the *Ubi7* gene promoter), and it is possible that the absence of expression variability is associated with this fact. The absence of expression variability within a single insertion site was demonstrated in a study on site-specific gene insertion into the tobacco genome using the Cre/Lox system [48]. However, the authors noted the silencing of transgenes, including mosaic silencing, among the resulting transgenic plants. The authors found that the level of transgene expression differed up to 10 times depending on the insertion site [48]. The absence of detectable transcription of the target gene in the presence of a detectable target protein for lines 38 and 4 may be due to the instability of the target protein mRNA synthesized in these lines. For these cell lines, the possibility of integration of target gene DNA fragments into random regions of the genome is not excluded, which could act as a trigger for the formation of short interfering RNAs that trigger its silence. The possibility of formation of a mosaic population of cells in plants with insertions of DNA fragments in inverse orientation to a target gene was shown by us earlier for tobacco plants [49]. Further work will be aimed at identifying factors associated with the variability of expression and accumulation of the target recombinant protein among site-specific events of integration of the target gene into regions of the genome with high transcriptional activity.

It should be emphasized that the integration of foreign genes in some cases is accompanied by a change in the expression of own plant genes located in the insertion region. It was found that insertions of transgenes can cause both large-scale chromosomal rearrangements and point mutations, as well as affect the epigenetic landscape of the surrounding chromatin [50]. Our analysis of the expression level of both plant genes surrounding the insertion site did not reveal any significant changes in their expression. The results obtained indicate that the site-specific insertion of the transgene did not disrupt the expression of the *HTR5* and 12 kDa subunit of microsomal peptidase genes, a change in the expression of which could reduce the viability of cells, as well as their ability to reproduce.

Even though the accumulation of dIFN protein in cell lines with targeted insertions into the target region of the *HTR5* gene does not statistically differ from the level of accumulation of dIFN protein in the group of lines with random integration of the transgene, it is among the knock-ins that lines 1–4 were identified, a monoclone of line 1, the accumulation of protein in which is more than 2% of the TSP. This cell line is of interest as a bioproducer for the accumulation of recombinant interferon gamma and can be used to reveal the potential for increasing the biosynthesis of recombinant protein by optimizing the conditions for cultivating plant cells and optimizing the composition of nutrient media.

## Figures and Tables

**Table 1:** The efficiency of delivery of the target gene to the region of the *HTR5* gene by three types of genetic constructs.

Genetic Construct	Number of Transformations	Number of Identified Knock-Ins	The Number of Progenitor Cell Lines for Monoclonal Lines With Knock-Ins	Number of Monoclonal Cell Lines Investigated
pIFN(H3.3).1	10	0	0	0
pIFN(H3.3).2	10	3 (1) *	2	4 (1.1–1.4) 2 (6.2; 6.3)
pIFN(H3.3).3	10	6 (3) *	3 (1) *	4 (38.3; 38.8; 38.22; 38.29) 5 (4.10; 4.15; 4.25; 4.27; 4.28)

* The number in brackets is the number of lines lost during the selection of monoclonal cell lines.

## Data Availability

Data sharing not applicable.

## Supplementary Materials

The following are available online at https://www.mdpi.com/article/10.3390/cells10082137/s1. Table S1: Oligonucleotides used in the work. Figure S1. Alignment of nucleotide sequences of PCR fragments obtained with primers UpH3.3_1 and Lo_plan3 from *A. thaliana* cell lines with knock-ins (lines 1, 6, 38 and 4) with DNA sequence of genetic construct pIFN(H3.3).3.

## Designations

LF and RF	left and right flanking sequences homologous to the intergenic region upstream the locus of *HTR5* gene;
P1	P-NOS promoter of *A. tumefaciens* nopaline synthase gene;
P2	CaMV35S promoter of cauliflower mosaic virus;
nptII	neomycin phosphotransferase II gene, which provides plant cell resistance to kanamycin;
S	DNA sequence encoding the leader signal of the carrot extensin gene, which ensures the transport of deltaferon into the apoplast;
dIFN	DNA sequence encoding the target dIFN protein;
GST	DNA sequence encoding a tag for affinity protein purification;
sgRNA	Cas9 endonuclease recognition sites homologous to the site in the intergenic region upstream the *A. thaliana HTR5* gene for excision of the construct from the plasmid in the cell.
