# Supplementary material for: Assessment of the Level of Accumulation of the dIFN Protein Integrated by the Knock-In Method into the Region of the Histone H3.3 Gene of Arabidopsis thaliana
Source: Cells. 2021 Aug 19;10(8):2137. doi: 10.3390/cells10082137 (PMC8394151; doi:10.3390/cells10082137)
Supplement: Supplementary file 1 [file cells-10-02137-s001.zip › cells-1287952-supplementary.pdf]

Supplementary

Table S1. Oligonucleotides used in the work.

Oligonucleotide	Nucleotide sequence	Gene or sequence	Received fragment size
1_gRNA_H Up	GATTCGGCCTAAACTAAATCCGAA	Sequence for guide RNA	
1_gRNA_H Lo	AAACTTCGGATTTAGTTTAGGCCG		
npt1	CGACGTTGTCACTGAAGCG	*nptII* marker gene	487
npt2	AAGCACGAGGAAGCGGTCAG		
Up_H3.3	TAGGCAACGATGGTAAAGCGGATT	Testing the site-specific integration	630/1048
Up_H3.3_1	AATCGCATAATCAAGAAAATCAAAACCC		
Lo_plan3	AGCCGAATAGCCTCTCCACCCAA		
dIFN 1	GATGTAGCGGATAATGGAACTCTTTT	*dIFN* target gene	478
dIFN 2	TGACTCCTTTTTCGCTTCCCTG		
VAP27-1_f	AACAAGACAAATAGCGATGTCCCA	12kDa gene of microsomal signal peptidase from *A. thaliana*	
VAP27-1_r	GCGATTAGATTCGTCCCGTGA		
VAP27-1_p	[R6G]CACCGTCTCCGGTTCAGAGAGAG[BHQ2]		
HIS3.3_f	CGGTGATCGTGAAACTACTGGAAT	Histone H3.3 gene from *A. thaliana*	
HIS3.3_r	CGGAAGATCATTCGGCGAAG		
HIS3.3_p	[ROX]CGCTCGATAGGTGGTACGAAATTAGG[BHQ2]		
PARP2_f	GGTGTTTCGTGTAGGTGGAGAGG	*PARP2*-gene from *A. thaliana*	
PARP2_r	CCTCTTGCTCTTTGATTCTTCCTTC		
PARP2_p	[Cy5]CTTGAAGAGGCTATCGCAGAAGACA[BHQ2]		
Up_sig.BglII(62)	CCCAGATCTATGGGAAGAATTGCTAGAGGC	Primers used to create constructs	
Lo_dIFN(60)	CTGGGATGCTCTTCGACCT		
ifn+Up_GST(60)	AGGTCGAAGAGCATCCCAGATGTCCCCTATACTAGGTTATT		
Lo_GST(GEX)stop A65I(66)	CCCGGTACCTTAACCACCAAACGTGGCTTGCCA		
Up_L_HIII	CCCAAGCTTCGAGCGATTCCAGTAGTTTCACGA		
Lo_L_Acc_Sfr	GGTACCAGAGTAATCTCGAGTTTATTTCAGCGATCCGCGACG		
Up_R_Sfr_Acc	CTCGAGATTACTCTGGTACCTTCGGATTTAGTTTAGGCCGTCACA		
Lo_R_Sal	CCCGTCGACCATCATCATCCTAAACGACGCCG		
Lo_A-B_pBI(58)	CGAAGGTACCGGATGGATCCCGTGTTCTCTCCAAATGAAAT		
Up_Xho-pBI(60)	AATCTCGAGGATCATGAGCGGAGAATTAAG		
Up_L_HIII_gRNA	CCCAAGCTTCGGCCTAAACTAAATCCGAACGGCGAGCGATTCCAGTAGTTTCACGA		
Lo_R_Sal_gRNA	CCCGTCGACGGCCTAAACTAAATCCGAACGGCATCATCATCCTAAACGACGCCG		
Up_Sal_gRNA-pBI(60):	CCCGTCGACCCGTTCGGATTTAGTTTAGGCCGGATCATGAGCGGAGAATTAAG		

Lo_A-B_pBI(58)	CGAAGGTACCGGATGGATCCCGTGTTCTCTCCA AATGAAAT		
Lo_Acc_gRNA_GST(60)	CGAAGGTACCCCGTTCGGATTTAGTTTAGGCCG ACCACCAAACGTGGCTTGCC		

Figure S1. Alignment of nucleotide sequences of PCR fragments obtained with primers UpH3.3_1 and Lo_plan3 from *A. thaliana* cell lines with knock-ins (lines 1, 6, 38 and 4) with DNA sequence of genetic construct pIFN(H3.3).3.

```
                           620                                               669
          1 A.th    (143)  GCTTTCTTGCACCAATTCAACGGTCCAGATCTAGCTTTACTGGTTTGACG
          6 A.th    (550)  GCTTTCTTGCACCAATTCAACGGTCCAGATCTAGCTTTACTGGTTTGACG
         38 A.th    (617)  GCTTTCTTGCACCAATTCAACGGTCCAGATCTAGCTTTACTGGTTTGACG
          4 A.th    (557)  GCTTTCTTGCACCAATTCAACGGTCCAGATCTAGCTTTACTGGTTTGACG
     pIFN(H3.3).3   (307)  GCTTTCTTGCACCAATTCAACGGTCCAGATCTAGCTTTACTGGTTTGACG

                           670                                               719
          1 A.th    (193)  AGGATCACCACTGCTACATAATTGATTGCTATTGGCTGAATACAAACCGT
          6 A.th    (600)  AGGATCACCACTGCTACATAATTGATTGCTATTGGCTGAATACAAACCGT
         38 A.th    (667)  AGGATCACCACTGCTACATAATTGATTGCTATTGGCTGAATACAAACCGT
          4 A.th    (607)  AGGATCACCACTGCTACATAATTGATTGCTATTGGCTGAATACAAACCGT
     pIFN(H3.3).3   (357)  AGGATCACCACTGCTACATAATTGATTGCTATTGGCTGAATACAAACCGT

                           720                                               769
          1 A.th    (243)  CGCGGATCGCTGAAATAAACCGTTCGGGATTTAGTTTAGGCCG--GATCA
          6 A.th    (650)  CGCGGATCGCTGAAATAAA-------------------GGCCG--GATCA
         38 A.th    (717)  CGCGGATCGCTGAAATAAAC----------------TCGACCG--GATCA
          4 A.th    (657)  CGCGGATCGCTGAAATAAAC----------------TCGACCG--GATCA
     pIFN(H3.3).3   (407)  CGCGGATCGCTGAAATAAAC----------------T----CGAGGATCA

                           770                                               819
          1 A.th    (291)  TGAGCGGAGAATTAAGGGAGTCACGTTATGACCCCCGCCGATGACGCGGG
          6 A.th    (679)  TGAGCGGAGAATTAAGGGAGTCACGTTATGACCCCCGCCGATGACGCGGG
         38 A.th    (749)  TGAGCGGAGAATTAAGGGAGTCACGTTATGACCCCCGCCGATGACGCGGG
          4 A.th    (689)  TGAGCGGAGAATTAAGGGAGTCACGTTATGACCCCCGCCGATGACGCGGG
     pIFN(H3.3).3   (437)  TGAGCGGAGAATTAAGGGAGTCACGTTATGACCCCCGCCGATGACGCGGG

                           820                                               869
          1 A.th    (341)  ACAAGCCGTTTTACGTTTGGAACTGACAGAACCGCAACGTTGAAGGAGCC
          6 A.th    (729)  ACAAGCCGTTTTACGTTTGGAACTGACAGAACCGCAACGTTGAAGGAGCC
         38 A.th    (799)  ACAAGCCGTTTTACGTTTGGAACTGACAGAACCGCAACGTTGAAGGAGCC
          4 A.th    (739)  ACAAGCCGTTTTACGTTTGGAACTGACAGAACCGCAACGTTGAAGGAGCC
     pIFN(H3.3).3   (487)  ACAAGCCGTTTTACGTTTGGAACTGACAGAACCGCAACGTTGAAGGAGCC

                           870                                         913
          1 A.th    (391)  ACTCAGCCGCGGGTTTCTGGAGTTTAATGAGCTAAGCACATACG
          6 A.th    (779)  ACTCAGCCGCGGGTTTCTGGAGTTTAATGAGCTAAGCACATACG
         38 A.th    (849)  ACTCAGCCGCGGGTTTCTGGAGTTTAATGAGCTAAGCACATACG
          4 A.th    (789)  ACTCAGCCGCGGGTTTCTGGAGTTTAATGAGCTAAGCACATACG
     pIFN(H3.3).3   (537)  ACTCAGCCGCGGGTTTCTGGAGTTTAATGAGCTAAGCACATACG
```
